# A Cross-Sectional Study of Exposure Factors Associated with Seropositivity for SARS-CoV-2 Antibodies during the Second Epidemic Wave among a Sample of the University of Corsica (France)

**DOI:** 10.3390/ijerph19041953

**Published:** 2022-02-10

**Authors:** Dorine Decarreaux, Julie Sevila, Shirley Masse, Lisandru Capai, Toscane Fourié, Paola Mariela Saba Villarroel, Abdennour Amroun, Elif Nurtop, Matthieu Vareille, Marie Pouquet, Thierry Blanchon, Xavier de Lamballerie, Rémi Charrel, Alessandra Falchi

**Affiliations:** 1 Laboratoire de Virologie, Université de Corse Pascal Paoli, UR7310 Bioscope, 20250 Corte, France; DECARREAUX_D@univ-corse.fr (D.D.); SEVILA_J@univ-corse.fr (J.S.); masse_s@univ-corse.fr (S.M.); capai_l@univ-corse.fr (L.C.); vareille_m@univ-corse.fr (M.V.); 2 INSERM, Sorbonne Université, Institut Pierre Louis d’Epidémiologie et de Santé Publique, IPLESP, 75012 Paris, France; marie.pouquet@iplesp.upmc.fr (M.P.); thierry.blanchon@iplesp.upmc.fr (T.B.); 3 Unité des Virus Émergents, Aix Marseille University, IRD 190, INSERM U1207, IHU Méditerranée Infection, 13005 Marseille, France; toscane.fourie@gmail.com (T.F.); marielasaba@gmail.com (P.M.S.V.); abdennour.amroun@inserm.fr (A.A.); enurtop@gmail.com (E.N.); xavier.de-lamballerie@univ-amu.fr (X.d.L.); remi.charrel@univ-amu.fr (R.C.)

**Keywords:** seroprevalence, antibodies, SARS-CoV-2, ELISA, seroneutralization

## Abstract

This study aimed to estimate the seroprevalence of severe acute respiratory syndrome coronavirus-2 (SARS-CoV-2) infection within the staff and student populations of the University of Corsica (France) during the second wave of the epidemic. Methods: A cross-sectional survey was conducted from 23 November 2020 to 31 January 2021. The participants underwent blood sampling using a fingerstick procedure and completed an anonymized questionnaire. Sera were tested for the presence of anti-SARS-CoV-2 IgG (ELISA-S) and, if positive, with an in-house virus neutralization test (VNT). Results: A total of 418 persons were included in the study. The overall seroprevalence was 12.8% (95% confidence interval (CI), 9.8–16.6%). A total of 15 (31%) of the 49 individuals who had a positive ELISA-S also had a positive VNT. Seropositivity was associated with living at the city campus during the week and on weekends (OR = 3.74 [1.40–12.00]), using public transportation/carpooling (OR = 2.00 [1.01–4.02]), and being in contact with a person who tested positive for SARS-CoV-2 (OR = 2.32 [1.20–4.40]). The main symptoms associated with seropositivity were “having had an acute respiratory infection” (OR = 3.05 [1.43–6.43]) and “experiencing loss of smell” (OR = 16.4 [5.87–50.7]). Conclusion: These results could be useful for SARS-CoV-2 prevention and control on university campuses.

## 1. Introduction

Managing schools and universities during the pandemic of the coronavirus disease 2019 (COVID-19) has been a global challenge [1]. Because of the proximity of many university students living in high-density housing and their extensive social networks compared with the general population, the potential for the rapid spread of SARS-CoV-2 in a university setting is of concern [2]. University students around the world have faced constraints such as the closure of campuses, the rapid and unplanned shift to online learning, and the introduction of gesture barriers, such as social distancing, the wearing of masks, and travel restrictions, aimed at reducing the transmission of the virus [3].

In France, universities were closed during the first (March through May 2020) and second (30 October 2020 to 15 December 2020) COVID-19 waves. The events of 2020 and 2021 have shown that university campuses could pose significant challenges to the control of the spread of SARS-CoV-2 [4]. A better understanding of SARS-CoV-2 circulation in the university population (faculty, staff, and students) and evaluation of the risk factors inherent to students regarding SARS-CoV-2 infection are critical for guiding universities.

To our knowledge, few studies have reported the seroprevalence of SARS-CoV-2 infection in universities [5,6,7,8,9]. In the current context, and given the current lack of knowledge in this field, we decided to set up a cross-sectional study to estimate the seroprevalence of SARS-CoV-2 within the staff and student populations of the University of Corsica (France), as well as the exposure factors and symptoms related to seropositivity during the second COVID-19 epidemic wave. The results of this study may provide insights about virus circulation in this population and can be used to inform decision making regarding university communities during viral spread.

## 2. Materials and Methods

### 2.1. Timeline

In France, the second wave of COVID-19 occurred from the week of 24 August (2020w35) to the week of 21 December (2020w52), 2020. During this second wave, schools and universities were kept open because of expanded testing resources and the implementation of health protocols (mask wearing, class spacing, physical distancing, contact tracing, quarantine, and self-isolation mandates). Schools and universities opened at the beginning of September 2020 and remained open until 18 December 2020, with the exception of a 2-week autumn break. During this second wave, students at the University of Corsica had the opportunity to attend face-to-face classes once or twice per week. All staff were present on the university campus, with teleworking facilities (at least once a week).

### 2.2. Study Design

This was a university-population-based cross-sectional study that included voluntary participation by the students and staff of the University of Corsica, Corte, France. Corte, the only city campus of Corsica, has 7000 inhabitants. The staff group included administrative staff, teachers, researchers, and PhD students. The purpose of this study was to estimate the seroprevalence of IgG antibodies against SARS-CoV-2 from capillary blood samples collected from 23 November 2020 to 31 January 2021. In relation to the sampling, the participants were asked to complete a questionnaire investigating sociodemographic data, comorbidities, symptoms, and past history of COVID-19, as well as behavioral factors. Both the blood samples and the questionnaires were anonymized.

### 2.3. Participants

All staff (*n* = 860) and students (*n* = 4442) of the University of Corsica were offered the possibility to participate in this study. They were invited by email to enroll in the study via their university mail on 23 November 2020. The students and staff were eligible to participate regardless of whether they had prior confirmed COVID-19 or COVID-19-related symptoms.

**Figure 1:**
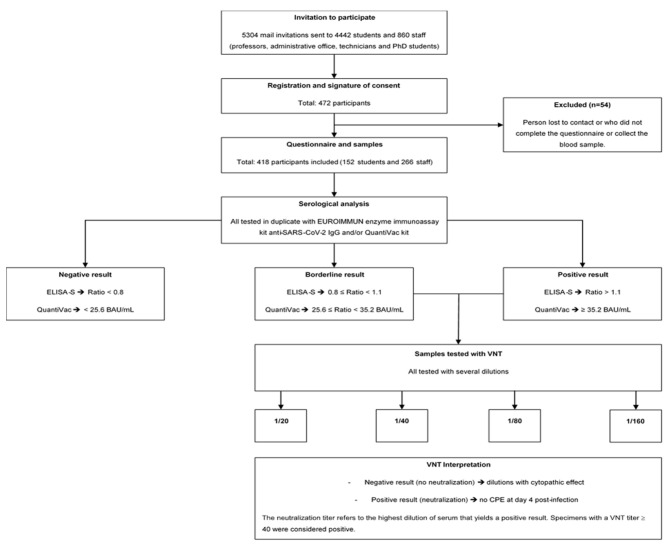
Flowchart of the study population.

Participants were included in the study after signing a consent form, and a unique identifier was assigned to them to guarantee their anonymity. Collections were carried out from 23 November 2020 to 31 January 2021. Because of the lockdown, which started on 29 October and ended on 15 December 2020, volunteers were offered to participate at home or on campus. Those who participated at home received a kit containing all the necessary components for an at-home capillary blood draw, including instructions, shipping materials, a self-use blood-collection device, a questionnaire, and a stamped self-addressed padded envelope to be returned to the Laboratory of Virology, UR7310 Bioscope of the University of Corsica. For participants who opted to participate at the university campus, blood samples were collected at the university’s health service center. The questionnaire was completed simultaneously. Participants who did not have a blood sample or did not complete the questionnaire were excluded from this study. The flowchart presented in Figure 1 describes the sample-inclusion process (Figure 1).

### 2.4. Outcomes

The primary outcome was the estimation of the seroprevalence of anti-SARS-CoV-2 IgG within the staff and student populations. Because the participants underwent serology testing between 23 November 2020 and 31 January 2021, the results reported here mainly reflect the circulation of SARS-CoV-2 since the summer of 2020, as IgG responses to the spike protein are stable for at least 6 months [10].

The secondary outcomes of the present study were as follows. (1) self-reported SARS-CoV-2 testing history, as assessed using the following question: “Since January 2020, have you ever tested positive for a SARS-CoV-2 infection?” (responses: “Yes”, “No”, “Don’t Know”); and (2) self-reported SARS-CoV-2 symptoms, as assessed using the following question: “Since January 2020, have you ever shown one of the following health statuses or symptoms?” (responses: “acute respiratory infection (sudden onset of fever or feeling of fever, with respiratory symptoms)”, “influenza-like illness (sudden onset of fever >39 °C, with muscle pain and respiratory symptoms)”, “loss of taste”, “loss of smell”, “nausea and/or vomiting, diarrhea”, “abdominal pain”, or “other symptoms (that participants could specify)”. More than one answer was possible).

### 2.5. Serological Analysis

Samples of capillary blood were obtained using a safety lancet on a cleansed finger puncture and collected into 0.8-mL tubes containing a coagulation activator and a serum separator. The tubes were centrifuged at 6000 rpm for 15 min and the resulting serum was stored at −20 °C until it was processed for serology.

Samples were tested in duplicate for the presence of anti-SARS-CoV-2 IgG using the EUROIMMUN enzyme immunoassay kit for the semiquantitative detection of IgG antibodies against the S1 domain of the viral spike protein (ELISA-S) (reference: EI 2606-9601 G; EUROIMMUN, Bussy-Saint-Martin, France). The assay has a specificity and a sensitivity of around 99.8% and 90.3%, respectively, according to the manufacturer’s data.

According to the manufacturer’s instructions, a result was considered borderline if the ratio was between 0.8 and <1.1, and positive if the sample ratio was 1.1. All samples with an ELISA-S ratio 0.8 and sufficient serum were then tested using the QuantiVac (IgG) kit (reference: El 2606-9601-10 G, EUROIMMUN, Bussy-Saint-Martin, France). Quantification of S1-specific IgG was performed using a 6-point calibration curve covering a range from 1 to 120 relative units (RU)/mL. By multiplication with a factor of 3.2, results in RU/mL were converted into standardized binding antibody units (BAU)/mL. A result of <25.6 BAU/mL was considered negative and a result of ≥35.2 BAU/mL was considered positive. In all ELISA-S positive and borderline samples, neutralizing antibodies were also detected using a virus neutralization test (VNT), as described previously [11]. VeroE6 cells cultured in 96-well microplates, 100 fifty-percent tissue culture infective dose (TCID_50_) of the SARS-CoV-2 strain BavPat1 (courtesy of Prof. Drosten, Berlin, Germany), and serial dilutions of serum (1/20–1/160) were used. Dilutions associated with a cytopathic effect (CPE) were considered negative (no neutralization), whereas those with no CPE at day 4 post infection were considered positive (complete neutralization). The neutralization titer refers to the highest dilution of serum that yields a positive result. Specimens with a VNT titer of 40 were considered positive (Figure 1).

### 2.6. Statistical Analysis

#### 2.6.1. Sample-Size Calculation

According to the overall IgG antibodies against SARS-CoV-2 seroprevalence observed in residual sera in mid-June in Corsica and at the end of October (clinical laboratory data) [12], a minimum sample size of 421 participants was calculated assuming an a priori 5% IgG antibodies against SARS-CoV-2 seroprevalence, a confidence in the estimate of 95%, a maximum allowable error in the prevalence of 1.5%, and a university population size of 5302 individuals.

#### 2.6.2. Analysis of Seroprevalence and Epidemiological Factors

The primary outcome was the estimation of the proportion of participants with a positive ELISA-S test. Baseline characteristics and exposures were presented as the number (%) for factors and the mean or median for numerical variables, as appropriate. Univariate analyses were performed to measure the effect of each variable on the serological status. The Wilcoxon rank sum test was used to compare two independent samples. Chi-squared and Fisher’s tests were used to compare differences between groups for categorical variables. Statistical significance was set at *p* < 0.05.

Seroprevalence was initially calculated as the proportion of participants who had a positive ELISA-S result. Subsequently, considering the limitations of the ELISA-S method, the seroprevalence estimate was also adjusted based on test sensitivity (90.3%) and specificity (99.8%) (reference: EI 2606-9601 G; EUROIMMUN, Bussy-Saint-Martin, France) using Epitools software (AUSVET, Canberra, Austria) [13].

Univariate logistic regression models were built to identify the potential factors associated with the serological status. Only variables that were close to significance (*p* ≥ 0.2) were retained for the multivariate analysis. Model selection was performed using the step Akaike information criterion. Two full models were built: one including the risk factors of exposure (such as type of accommodation), and the other including factors representing the consequence of the exposure (such as the following symptoms: acute respiratory infection, influenza-like illness, loss of taste, and loss of smell). For the first type of model, seven variables (education level, residential lifestyle, accommodation type, case contact since January 2020, use of public transportation/carpooling, social interaction level, and worry about own health) were selected to compose the full model. For the second type of model, four variables (acute respiratory infection, influenza-like illness, loss of taste, and loss of smell) were selected to compose the full model. Odds ratios (ORs) and 95% confidence intervals (CIs) were used to explore factors potentially associated with a positive ELISA-S result.

Statistical analyses were performed using R software, version 4.0.4 (R Fundation, Vienna, Austria) [14].

### 2.7. Ethics

The study protocol was approved on 12 November 2020, by the ad hoc ethics committee (Comité de Protection des Personnes #2020-A00711-38). All data and questionnaires were checked and validated by the data protection officer of the University of Corsica. All participants in this study were volunteers and unpaid. The entire university population was informed that the samples would be used for epidemiological studies via an information letter. Written informed consent was required for the use of the data in the analyses.

## 3. Results

### 3.1. Sociodemographic Data Description

From 23 November 2020 to 31 January 2021, a total of 418 persons (266 staff members and 152 students) who filled out a questionnaire and had full serology were included in the study (Figure 1). The baseline characteristics of the participants are summarized in Table 1. Among the 418 participants, 65% (*n* = 271) were female and the median age was 31 (IQR: 22–44) years. Half of the participants (50%; *n* = 209) were postgraduate. Of the 418 participants, 44% (*n* = 184) lived in Corte (the city hosting the university campus) and about half of the population lived in apartments (57%; *n* = 223). Finally, 28% (*n* = 117) of the participants had at least one comorbidity (Table 1).

### 3.2. Symptoms and SARS-CoV-2 History

As shown in Table 1, 36% (*n* = 149) of the participants declared having had COVID-19-related symptoms since 1 January 2020, and 6.5% (*n* = 27) of them declared having previously tested positive (PCR test) for SARS-CoV-2 since 1 January 2020. Among the 27 confirmed cases, 5.3% (*n* = 22) declared having had at least one symptom related to virologically confirmed SARS-CoV-2.

Twenty-six percent (*n* = 109 out of 418) of the participants reported having had at least one contact with a confirmed COVID-19-positive individual since 1 January 2020. Among those 109 participants, 28% (*n* = 31) had more than one contact episode. The majority of contacts consisted of interactions with coworkers or classmates (45%; *n* = 50 out of 109), followed by contact with family members (29%; *n* = 32 out of 109) and friends (26%; *n* = 29 out of 109).

### 3.3. Seroprevalence of IgG Antibodies against SARS-CoV-2

Overall, 49 of the 418 participants (11.7%; 95% CI, 8.8–15.3%) tested positive on ELISA-S, with similar values recorded between students (10.5%) and staff members (12.4%) (*p* = 0.57) (Table 1). According to the sensitivity and specificity of the ELISA-S test, true seroprevalence was estimated at 12.8% (95% CI, 9.8–16.6%) (Table 1).

The serological status and the self-reported positive SARS-CoV-2 test results according to the presence or absence of symptoms are presented in Table 2.

Among the 49 participants with a positive ELISA-S result, 63.3% (*n* = 31) declared having experienced at least one COVID-19 symptom since 1 January 2020. The symptoms experienced by participants that were most associated with a positive ELISA-S result (*p* < 0.001) were loss of smell (9.4%; *n* = 14 out of 149) and loss of taste (7.4%; *n* = 11 out of 149).

### 3.4. Quantitative Results

**Figure 2:**
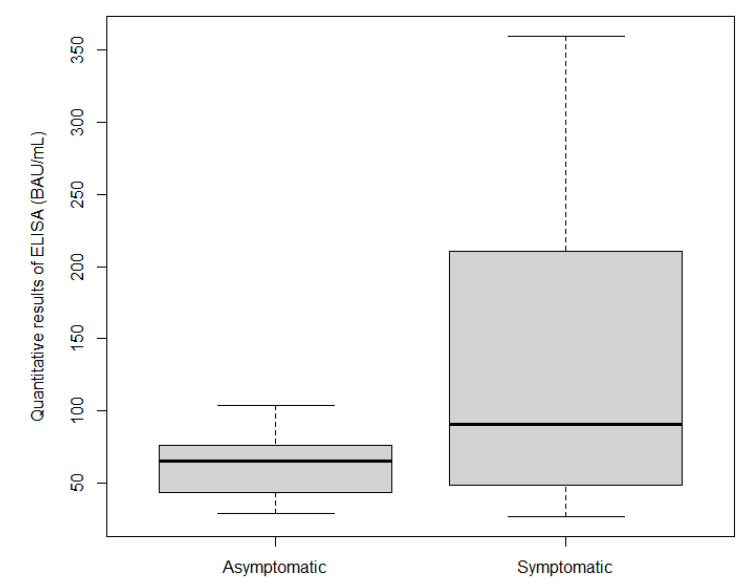
Box plot of quantitative ELISA results according to symptoms.

The IgG antibody levels against SARS-CoV-2 in 77.5% (*n* = 38 out of 49) of the ELISA-S positive samples were measured quantitatively using QuantiVac ELISA-S. The mean IgG antibody level of symptomatic individuals (124.37 BAU/mL) was slightly higher than, but not significantly different from, that of asymptomatic individuals, at 62.33 BAU/mL (*p* = 0.08175) (Figure 2).

### 3.5. Seroneutralization Results

The seroneutralization results obtained for the study population are presented in Table 3. Among the 49 individuals who had a positive ELISA-S result, 31% (*n* = 15) had a positive result on the VNT. Among the 15 participants who were seroneutralization positive, 46.7% (*n* = 7) had a VNT titer of 40, 33.3% (*n* = 5) had a VNT titer of 80, and 20.0% (*n* = 3) had a VNT titer of 160.

The serum neutralization titers did not differ significantly between students and staff or according to age and gender. Individuals who had developed symptoms or reported a confirmed COVID-19 positive result since January 2020 had higher neutralizing antibody titers than did asymptomatic individuals (*p* = 0.024 and *p* < 0.001, respectively) (Table 3).

### 3.6. Sociobehavioral Characteristics of the Population

The sociobehavioral characteristics of the study population are presented in Supplementary Table S1. Outside of the lockdown period (between 11 May and 29 October 2020), 60% (*n* = 249) of the participants reported never using public transportation or carpooling. Regarding social interactions outside of a work, home, and public transport context (e.g., at the supermarket, the gym, or a bar), 44% (*n* = 184) of the participants reported having a few of these interactions several times per week. Regarding the adherence to barrier behaviors, 68% (*n* = 284) of the participants mentioned adopting the correct actions and behaviors to protect themselves and others from SARS-CoV-2. At least 30% (*n* = 152) of the respondents reported being a little concerned about the consequences of SARS-CoV-2 infection on their own health, whereas 43% (*n* = 178) were very concerned about the health of individuals close to them.

### 3.7. Association between Factors and Seropositivity

The results of the univariate and multivariate analyses are shown in Supplementary Table S2 and Table 4, respectively. The following factors were associated with seropositivity: living in Corte (OR = 3.74 [1.40–12.00]; *p* = 0.014), using public transportation/carpooling (OR = 2.00 [1.01–4.02]; *p* = 0.048), having a case contact since January 2020 (OR = 2.32 [1.20–4.40]; *p* = 0.012), the presence of an acute respiratory infection (OR = 3.05 [1.43–6.43]; *p* = 0.005) and loss of smell (OR = 16.4 [5.87–50.7]; *p* < 0.001). Living in a house (OR = 0.36 [0.15–0.77]; *p* = 0.013) was a protective factor against prior SARS-CoV-2 infection (Table 4).

## 4. Discussion

This study revealed that this university community showed a significantly higher seroprevalence relative to the general population. In fact, this cross-sectional study of IgG antibodies against SARS-CoV-2 seroprevalence, which was carried out from 23 November 2020 to 31 January 2021 at the University of Corsica (France), showed that 12.8% of the participants who had a positive ELISA-S result among the students and staff had evidence of prior infection, with at least 30% of them showing neutralizing antibodies. Notably, this rate, which was similar between the students and faculty staff, was significantly higher than the 8.04% (95% CI, 7.24–8.84%) observed in the residual sera of the Corsican general population during the same period, as assessed using the same serological assays [15]. In this study, we reported that almost 40% of the participants who were positive on ELISA-S reported no symptoms since January 2020. We also observed an increased risk of seropositivity concomitant with a wide range of clinical and sociodemographic factors. As IgG responses to the spike protein are stable for at least 6–8 months [10], the results reported here mainly reflected the circulation of SARS-CoV-2 among the participants since the summer of 2020.

However, our seropositivity rate was in the lower range of that observed in a cross-sectional UK study of SARS-CoV-2 antibody seroprevalence among the university student population, which reported a rate ranging between 7.6% and 29.7% across the five participating UK universities during the second wave of the epidemic (December 2020) [16].

We investigated the association between demographical, clinical, and lifestyle factors and seropositivity. In the present study, participants living in Corte (city campus) during the week and on weekends were at greater risk than were participants living outside Corte during the week and outside Corte on weekends. Because Corte is a small town (approximately 7000 inhabitants), we can assume that contacts between people are more frequent, as they come into contact more often in the same indoor places (e.g., there is only one main supermarket in the city). Furthermore, we know that SARS-CoV-2 can remain airborne for up to three hours and survive on surfaces [17]. In agreement with previous studies [18,19], we observed that participants living in houses were at a lower risk of infection than were participants living in apartments. It can be hypothesized that single-family homes allow more frequent access to the outdoors than do apartments. Moreover, apartments have common areas (stairwells, elevators, garbage rooms, and mailbox rooms, etc.), which do not always allow good ventilation and encourage people to meet. Similar to a nationwide French case–control study carried out during the second wave [20], we identified public transportation/carpooling as a risk of SARS-CoV-2 infection. Public transportation and the mobility of the population facilitate the rapid transmission of viruses, particularly during the SARS-CoV-2 pandemic [21,22].

In addition, we investigated the association between seropositivity and a history of SARS-CoV-2. Among the participants, a positive ELISA-S result was significantly associated with case contact or with having reported testing positive for SARS-CoV-2 since January 2020. The majority of case contacts reported by the participants consisted of interactions with coworkers or classmates, which suggests that the university community may favor the transmission of and contamination with SARS-CoV-2. This is in line with previous studies showing that close contact with people infected with COVID-19 increases viral transmission [23].

Furthermore, seroprevalence was strongly associated with at least one of the symptoms self-reported since 1 January 2020. The main symptoms associated with seropositivity were “having had an acute respiratory infection” and “experiencing loss of smell”. As reported previously [24], anosmia may be the first presenting symptom, preceding the occurrence of other COVID-19 symptoms with less specificity, such as a cough and fever [25]. In addition, we found that individuals who had reported symptoms since January 2020 had a significantly greater titer of IgG antibodies against SARS-CoV-2 and of neutralizing antibodies than did those who had not reported symptoms. This agrees with recent reports indicating that asymptomatic individuals with COVID-19 mount a neutralizing humoral response that is lower than that observed in symptomatic persons or hospitalized patients with COVID-19 [26].

Interestingly, almost 40% of the participants who were positive on ELISA-S reported no symptoms since January 2020. This is in agreement with current data suggesting that infected persons without symptoms—including both presymptomatic and asymptomatic persons—account for more than 40% of all SARS-CoV-2 transmission [27,28,29]. This result shows that COVID-19 control strategies have to consider the prevalence and transmission risk of asymptomatic SARS-CoV-2 infection.

This study had several limitations. The selected university community may not be representative; indeed, our population is composed mainly of university staff (composed of research professors, administrative staff, and PhDs) since this is the group that responded most favorably to the call for participation. It is a specific population and data were obtained through a self-reported questionnaire; however, it is reasonable to assume that the awareness and memory of symptoms that may be related to COVID-19 have increased, leading to a lower estimate of the asymptomatic fraction. Furthermore, these results cannot be extrapolated directly to the general population. In turn, this study had several strengths. In particular, it was conducted among a university community for which data were scarcely available. In addition, serological samples were collected during the second wave of intense SARS-CoV-2 circulation. Finally, several serological methods were used to improve the interpretation of the seroprevalence results.

## 5. Conclusions

This study revealed that the university community showed a significantly higher seroprevalence relative to the general population and that a large proportion had no significant symptoms related to COVID-19. The results highlight the setting in which adherence to measures including hand and respiratory hygiene, physical distancing, mask wearing, and the ventilation of indoor environments is critical to control the spread of SARS-CoV-2 viruses. It would be interesting to carry out a follow-up antibody quantification to evaluate the natural immunity and/or vaccination of the university population over time.

## Figures and Tables

**Table 1:** Sociodemographic and clinical characteristics of the academic population.

Characteristic	Overall, *n* = 418 ^1^	Academic Population		*p*-Value ^2^
		Student, *n* = 152 ^1^	University Staff, *n* = 266 ^1^	
**Mean age (min; max)**	33.8 (17; 64)	21.5 (17; 44)	40.8 (20; 64)	<0.001
**Age group**				<0.001
<20 years	54 (13%)	54 (36%)	0 (0%)	
[20–29 years]	143 (34%)	91 (60%)	52 (20%)	
[30–39 years]	82 (20%)	4 (2%)	78 (29%)	
[40–49 years]	73 (17%)	3 (2%)	70 (26%)	
>50 years	66 (16%)	0 (0%)	66 (25%)	
**Gender** Female	271 (65%)	97 (64%)	174 (65%)	0.742
**Education level**				<0.001
High school level and under	75 (18%)	49 (32%)	26 (10%)	
Bachelor’s degree	134 (32%)	75 (49%)	59 (22%)	
Master’s degree	122 (29%)	28 (18%)	94 (35%)	
Over master’s degree	87 (21%)	0 (0%)	87 (33%)	
**Accommodation type**				<0.001
Halls of residence	28 (7.1%)	28 (22%)	0 (0%)	
Apartment	223 (57%)	83 (65%)	140 (53%)	
House	141 (36%)	17 (13%)	124 (47%)	
Unknown	26	24	2	
Residential district *				<0.001
Corte	184 (44%)	23 (15%)	161 (61%)	
Corte/Elsewhere	137 (33%)	95 (62%)	42 (16%)	
Elsewhere	97 (23%)	34 (22%)	63 (24%)	
Chronic diseases **	117 (28%)	34 (22%)	83 (31%)	0.048
Unknown	2	0	2	
COVID-19 symptoms at any time since January 2020 ***	149 (36%)	66 (43%)	83 (31%)	0.012
**Case contact since January 2020**	109 (26%)	50 (33%)	59 (22%)	0.016
**Confirmed SARS-CoV-2 cases since January 2020 (self-report)**	27 (6.5%)	11 (7.2%)	16 (6.0%)	0.62
**COVID-19 symptoms of SARS-CoV-2 confirmed cases**	22 (5.3%)	9 (5.9%)	13 (4.9%)	0.6
**Serological status (ELISA-S)**				0.57
Presence Ac IgG-S Adjusted according to test sensitivity and specificity	49 (11.7%) 49 (12.8%)	16 (10.5%) 16 (11.5%)	33 (12.4%) 33 (13.5%)	

^1^ Mean (SD); *n* (%). ^2^ Wilcoxon rank sum test; Pearson’s chi-squared test. * Three profiles were observed: people who lived in Corte year-round (Corte), those who lived in Corte during the week and returned to their family home on weekends (Corte/Elsewhere), and those who lived outside of Corte during the week and on weekends (Elsewhere). ** Chronic diseases (obesity, diabetes, hypertension, heart disease, asthma, pulmonary pathology, liver disease, chronic neurological disease, chronic renal failure, rheumatological disease, cancer, and immunosuppressive disease). *** Symptoms presented during the year 2020 (acute respiratory infection, influenza-like illness, loss of taste, loss of smell, nausea/vomiting, diarrhea, abdominal pain, etc.).

**Table 2:** Serological and reported positive RT-qPCR/antigen test results according to declared symptomatology.

Characteristic	Reported No Symptoms at Any Time Since 1 January 2020	Reported Experiencing Symptoms at Any Time Since 1 January 2020	*p*-Value ^1^
Positive ELISA-S (*n* = 49)	18 (36.7%)	31 (63.3%)	<0.001
Positive RT-qPCR/ antigen test (*n* = 27)	5 (18.5%)	22 (81.5%)	<0.001
Positive RT-qPCR and positive ELISA-S (*n* = 19)	3 (15.8%)	16 (84.2%)	<0.001

^1^ Pearson’s chi-squared test; Wilcoxon rank sum exact test.

**Table 3:** Main characteristics of the participants with positive seroneutralization results.

Characteristic	Overall, *n* = 49 ^1^	Seroneutralization Results		*p*-Value ^2^
		Negative, *n* = 34 ^1^	Positive, *n* = 15 ^1^	
**Function**				0.20
Student	16 (33%)	9 (26%)	7 (47%)	
University staff	33 (67%)	25 (74%)	8 (53%)	
**Mean age (years)**	32.20 (12.5)	33.53 (12.2)	29.20 (13.1)	0.13
**Gender**				0.60
Female	30 (61%)	20 (59%)	10 (67%)	
**Symptoms since January 2020**				0.024
Asymptomatic	18 (37%)	16 (47%)	2 (13%)	
Symptomatic	31 (63%)	18 (53%)	13 (87%)	
**Mean quantification (BAU/mL)** Unknown	108.0 (85.5) 11	73.6 (52.1) 11	160.9 (100.5) 0	0.002
**Positive COVID-19 test since January 2020**	19 (39%)	6 (18%)	13 (87%)	<0.001

^1^ Mean (SD); *n* (%). ^2^ Fisher’s exact test; Wilcoxon rank sum test; Pearson’s chi-squared test; Wilcoxon rank sum exact test.

**Table 4:** Multivariate analysis of the characteristics associated with SARS-CoV-2 seropositivity in the university population.

Characteristic	Description of Selected Variables (Univariate Analysis)				Effect of Selected Variables (Multivariate Analysis)	
	Overall, *n* = 390 ^1^	ELISA Results		*p*-Value ^2^	Odds Ratio [95% CI]	*p*-Value ^2^
		Negative, *n* = 342 ^1^	Positive, *n* = 48 ^1^			
**Residential lifestyle**				0.10		0.018
Elsewhere	86 (22%)	81 (55%)	5 (10%)		-	-
Corte/Elsewhere	123 (32%)	107 (31%)	16 (33%)		1.80 [0.64–5.87]	0.300
Corte	181 (46%)	154 (45%)	27 (56%)		3.74 [1.40–12.00]	0.014
**Accommodation type**				0.016		0.028
Apartment	222 (57%)	186(54%)	36 (75%)		-	-
House	140 (36%)	131 (38%)	9 (19%)		0.36 [0.15–0.77]	0.013
University residence	28 (7%)	25 (8%)	3 (6%)		0.64 [0.14–2.08]	0.500
**Use of public transportation/carpooling**	154 (39%)	131 (38%)	23 (48%)	0.200	2.00 [1.01–4.02]	0.048
**Case contact since January 2020**	97 (25%)	77 (23%)	20 (42%)	0.004	2.32 [1.20–4.40]	0.012
**Acute respiratory infection**	67 (16%)	47 (13%)	20 (41%)	<0.001	3.05 [1.43–6.43]	0.005
**Loss of smell**	20 (4.8%)	6 (1.6%)	14 (29%)	<0.001	16.4 [5.87–50.7]	<0.001

^1^*n* (%).^2^ Chi-squared test with Rao & Scott’s second-order correction.

## Data Availability

Not applicable.

## Supplementary Materials

The following supporting information can be downloaded at: https://www.mdpi.com/article/10.3390/ijerph19041953/s1, Table S1: Sociobehavioral characteristics of the academic population; Table S2: Univariate analysis of the characteristics associated with SARS-CoV-2 seropositivity in the university population.
