# Supplementary material for: A Cross-Sectional Study of Exposure Factors Associated with Seropositivity for SARS-CoV-2 Antibodies during the Second Epidemic Wave among a Sample of the University of Corsica (France)
Source: Int J Environ Res Public Health. 2022 Feb 10;19(4):1953. doi: 10.3390/ijerph19041953 (PMC8872171; doi:10.3390/ijerph19041953)
Supplement: Supplementary file 1 [file ijerph-19-01953-s001.zip › ijerph-1565948-supplementary.pdf]

Supplementary Table S1. Sociobehavioral characteristics of the academic population.

Characteristic	Overall, n = 418[1]	Academic population		P-value[2]
		Student, n = 152[1]	University staff, n = 266[1]	
**Frequency of public transportation/carpooling (excluding containment)**				<0.001
Never	249 (60%)	63 (41%)	186 (70%)	
Occasionally	118 (28%)	52 (34%)	66 (25%)	
Several times a week	38 (9.1%)	28 (18%)	10 (3.8%)	
Every day	13 (3.1%)	9 (5.9%)	4 (1.5%)	
**Frequency of social interaction (excluding containment)**				0.002
Never/Occasionally	122 (29%)	31 (21%)	91 (34%)	
Several times a week	184 (44%)	67 (44%)	117 (44%)	
Every day	110 (26%)	53 (35%)	57 (22%)	
Unknown	2	1	1	
**Respect for barrier gestures**				<0.001
No/Mostly no	12 (2.9%)	10 (6.6%)	2 (0.8%)	
Mostly yes	121 (29%)	49 (32%)	72 (27%)	
Yes	284 (68%)	92 (61%)	192 (72%)	
Unknown	1	1	0	
**Worry about own health**				0.003
Not at all	80 (19%)	41 (27%)	39 (15%)	
A little	152 (36%)	59 (39%)	93 (35%)	
Moderately	132 (32%)	36 (24%)	96 (36%)	
A lot	53 (13%)	15 (9.9%)	38 (14%)	
Unknown	1	1	0	
**Worry about the health of individual close to them**				0.30
Not at all	9 (2.2%)	5 (3.3%)	4 (1.5%)	
A little	97 (23%)	37 (25%)	60 (23%)	
Moderately	133 (32%)	41 (27%)	92 (35%)	
A lot	178 (43%)	68 (45%)	110 (41%)	
Unknown	1	1	0	

[1] n (%).

[2] Fisher's exact test; Pearson's chi-squared test.

Supplementary Table S2. Univariate analysis of the characteristics associated with SARS-CoV-2 seropositivity in the university population.

Characteristic	Overall, n = 418[1]	ELISA results: Negative, n = 369[1]	ELISA results: Positive, n = 49[1]	Odds ratio [95%CI]	P-value[2]
**Education level**					0.119
High school level and under	75 (18%)	69 (19%)	6 (12%)	-	-
Bachelor's degree	134 (32%)	112 (30%)	22 (45%)	2.36 [0.92–6.42]	0.10
Master's degree	122 (29%)	107 (29%)	15 (31%)	1.61 [0.62–4.73]	0.30
Over master's degree	87 (23%)	81 (22%)	6 (12%)	0.85 [0.25–2.86]	0.80
**Residential life style**					0.110
Elsewhere	97 (23%)	91 (25%)	6 (12%)	-	-
Corte/Elsewhere	137 (33%)	121 (33%)	16 (33%)	2.01 [0.79–5.80]	0.20
Corte	184 (44%)	157 (43%)	27 (55%)	2.61 [1.10–7.23]	0.04
**Accommodation type**					0.015
Apartment	223 (57%)	187(54%)	36 (75%)	-	-
House	141 (36%)	132 (38%)	9 (19%)	0.35 [0.16–0.73]	0.008
Halls of residence	28 (7%)	25 (8%)	3 (6%)	0.62 [0.14–1.91]	0.5
Unknown	26	25	1		
**Use of public transportation/carpooling**	169 (40%)	145 (39%)	24 (49%)	1.48 [0.81–2.71]	0.194
**Social interactions level**					0.087
Never/Occasionally	122 (29%)	108 (29%)	14 (29%)	-	-
Several times a week	184 (44%)	168 (46%)	16 (33%)	0.73 [0.34–1.59]	0.4
Every day	110 (27%)	91 (25%)	19 (39%)	1.61 [0.77–3.46]	0.2
Unknown	2	2	0		
**Worry about own health**					0.147
Not at all - A little	232 (56%)	200 (54%)	32 (65%)	-	-
Moderately - A lot	185 (44%)	168 (46%)	17 (35%)	0.63 [0.33–1.17]	0.2
Unknown	1	1	0		
**Case contact since January 2020**	109 (26%)	88 (24%)	21 (43%)	2.39 [1.28–4.42]	0.004
**Symptoms at any time since January 2020**	149 (36%)	118 (32%)	31 (63%)	3.66 [1.99–6.94]	<0.001
Acute respiratory infection	67 (16%)	47 (13%)	20 (41%)	4.72 [2.45–9.01]	<0.001
Influenza-like illness	48 (11%)	34 (9.2%)	14 (29%)	3.94 [1.89–7.96]	<0.001
Loss of taste	16 (3.8%)	5 (1.4%)	11 (22%)	21.1 [7.25–70.1]	<0.001
Loss of smell	20 (4.8%)	6 (1.6%)	14 (29%)	24.2 [9.09–72.3]	<0.001
**Confirmed SARS-CoV-2 cases since January 2020 (self-report)**	27 (6.5%)	8 (2.2%)	19 (39%)	28.6 [11.9–74.7]	<0.001

[1] n (%).

[2] Chi-squared test with Rao & Scott's second-order correction.
